# Successful pneumonectomia of a patient with squamous cell carcinoma of lung operated by median sternotomy and anterior approach due to suspicion of left main pulmonary artery invasion and possible need for cardiopulmonary bypass

**DOI:** 10.1186/1749-8090-10-S1-A221

**Published:** 2015-12-16

**Authors:** Kenan Can Ceylan, Ufuk Yetkin, Köksal Dönmez, Örs Şeyda Kaya, Ali Gürbüz

**Affiliations:** 1 Izmir Suat Seren Hospital, Dept. of Thoracic Surgery, Gaziler Cad. No: 331, 35110 İzmir, Turkey; 2 Department of Cardiovascular Surgery, Katip Celebi University Izmir Ataturk Training and Research Hospital, Izmir, Turkey

## Background/Introduction

Peripheral invasion of cancers may require multidisciplinary approach for increasing safety and efficacy of curative surgical interventions.

## Aims/Objectives

Our case was 58 year-old male patient. He was treated with chemo/radiotherapy for six times due to bronchial carcinoma in a different clinic for 9 months.

## Method

Thorax CT revealed a lesion of 40x43 mm enveloping left main pulmonary artery and bronchi in left hilus. Chest and Cardiovascular surgery council planned an intervention consisting of left pneumonectomy across pericardium, because of suspicion of left main pulmonary artery invasion, vascular graft interposition to left main pulmonary artery and emergent cardiopulmonary bypass if necessary.

## Results

Median sternotomy was performed after intubation with double lumen endotracheal tube and general anesthesia. Adhesions were removed after entering left pleural cavity. A tumoral lesion of 4x3x3 cm, advancing to pericardium was localized centrally at upper lobe's anterior segment and it was invading left main pulmonary artery. Pericardium was explored for evaluating invasion of intrapericardial main pulmonary artery. Intrapericardial ligation was decided to be enough for operation. Intrapericardial Left main pulmonary artery was ligated twice and dissected after transfixation sutures were placed. Superior and inferior pulmonary veins were dissected and transected by two vascular stapler. Left main bronchi was dissected and transected by stapler. Sixth and ninth mediastinal lymph nodes were dissected in mediastinum. Optimal controls for bleeding and air leak was achieved. Histopathological examination of pleural fluid was reported as benign cytology. Left pneumonectomy material was reported as squamous cell carcinoma (non-keratinized pT2b, N2) with middle degree differentiation localized in hilus, which had bronchial obstructive lesion and peribronchial infiltration characteristics. Perineural and vascular invasion was not observed. Metastasis was observed in two peribronchial lymph nodes and 14 perihiler and bronchial lymph nodes were free from metastasis. Adjuvant chemotherapy was planned in postoperative Surgery-Oncology council.

## Discussion/Conclusion

Risk evaluation of oncologic patients and Multidisciplinary approach to patients who are candidates for surgery may increase curability and survival rates.

## Consent

Written informed consent was obtained from the patient for publication of this Case report and any accompanying images. A copy of the written consent is available for review by the Editor-in-Chief of this journal.

